# 200 Reflections on Ireland’s Physical Literacy Journey

**DOI:** 10.1093/eurpub/ckae114.023

**Published:** 2024-09-26

**Authors:** Sinead Connolly, Elaine Murtagh, Wesley O’Brien, Hannah Goss

**Affiliations:** Ulster University, Ireland; University Of Limerick, Ireland; University College Cork, Ireland; Dublin City University, Ireland

## Abstract

**Purpose:**

Recognition of the importance of physical literacy (PL) in promoting lifelong physical activity, health, and well-being stems from the early beginnings of work in the early 2000s. This paper aims to explore the development of physical literacy in Ireland and Northern Ireland over the past 20 years.

**Policy Description:**

Since the early 2000s, on the Island of Ireland, education, sport bodies, local authorities and researchers were progressive and innovative in developing a range of physical literacy (PL) projects. Work was initially disjointed. In 2009, collaboration between Sport Ireland and Sport NI established the Lifelong Involvement in Sport and Physical Activity framework and the cross sectoral PL Task and Finish Group marked a more strategic, evidenced-based and cooperative approach to PL developments. More recently, several university-based PL research initiatives have developed and evaluated programmes, including Y-Path, Project FLAME, and Moving Well-Being Well. The Active School Programme and Curriculum Sport Programmes in Northern Ireland gained traction. Unfortunately, PL developments continue to be hampered by a lack of cross-sectoral coordination and sustainable funding, leading to some flagship developments being stalled. Latterly, attempts have been made to integrate PL into national and regional policies related to education, health, and sport e.g. Active Living - the new Sport and Physical Activity Strategy for Northern Ireland (Department of Communities NI) and The Healthy Ireland – ‘Get Ireland Active’, National Activity Plan for Ireland. Most recently, a key milestone was the development of the All-Island Physical Literacy Consensus Statement, the first of its kind in Europe and uniquely covers two jurisdictions. This statement provides opportunities for implementation at an individual, organisational, and systemic levels to promote effective advocacy and development of PL in Ireland and Northern Ireland.

**Conclusions:**

By examining the background, early policy, research, programmes, the process of developing a consensus statement, the current position in policy, and the challenges for implementation, this paper aims to contribute to the understanding of physical literacy promotion in the region. It also offers valuable insights and recommendations for future efforts to enhance physical literacy and improve the health and well-being of individuals in Ireland and Northern Ireland.

